# Identification of Polo-like kinases as potential novel drug targets for influenza A virus

**DOI:** 10.1038/s41598-017-08942-7

**Published:** 2017-08-17

**Authors:** Marie O. Pohl, Jessica von Recum-Knepper, Ariel Rodriguez-Frandsen, Caroline Lanz, Emilio Yángüez, Stephen Soonthornvacharin, Thorsten Wolff, Sumit K. Chanda, Silke Stertz

**Affiliations:** 1 Institute of Medical Virology, University of Zurich, Winterthurerstrasse 190, 8057 Zurich, Switzerland; 2 Life Sciences Zurich Graduate School, ETH and University of Zürich, 8057 Zurich, Switzerland; 3 Immunity and Pathogenesis Program, Infectious and Inflammatory Disease Center, Sanford Burnham Prebys Medical Discovery Institute, 10901 North Torrey Pines Road, La Jolla, CA 92037 USA; 4 Unit 17, Influenza and Other Respiratory Viruses, Robert Koch Institute, 13353 Berlin, Germany

## Abstract

In recent years genome-wide RNAi screens have revealed hundreds of cellular factors required for influenza virus infections in human cells. The long-term goal is to establish some of them as drug targets for the development of the next generation of antivirals against influenza. We found that several members of the polo-like kinases (PLK), a family of serine/threonine kinases with well-known roles in cell cycle regulation, were identified as hits in four different RNAi screens and we therefore studied their potential as drug target for influenza. We show that knockdown of PLK1, PLK3, and PLK4, as well as inhibition of PLK kinase activity by four different compounds, leads to reduced influenza virus replication, and we map the requirement of PLK activity to early stages of the viral replication cycle. We also tested the impact of the PLK inhibitor BI2536 on influenza virus replication in a human lung tissue culture model and observed strong inhibition of virus replication with no measurable toxicity. This study establishes the PLKs as potential drug targets for influenza and contributes to a more detailed understanding of the intricate interactions between influenza viruses and their host cells.

## Introduction

Influenza is a respiratory febrile disease in humans that is caused by influenza A and B viruses. In winter season epidemics of influenza are common and cause a substantial disease burden but also high economical losses due to sick days. Influenza A viruses (IAVs) can also cause pandemic outbreaks which are rare but can come with devastating consequences. The development of treatment and prevention options for influenza is therefore of high priority.

Currently, we have vaccines and antiviral drugs available, but both come with limitations. While the available vaccines are our best option to prevent influenza, a major problem is that they only provide protection against a small number of virus strains. If a novel virus strain arises, or there is a mismatch between the vaccine strain and the circulating viruses protection provided by vaccines is limited^1^. Therefore, it is important to have both, a number of antivirals available for the treatment of influenza patients, and also for prophylactic administration. Unfortunately, only one of the two approved drug classes for influenza can still be used: circulating strains are resistant to the inhibitors of the viral ion channel M2, the so-called adamantanes^2, 3^, and we are therefore left with the inhibitors of the viral neuraminidase. Novel antivirals are urgently sought and many research efforts are dedicated to the development of additional drugs^4^.

Classically, antivirals, such as the influenza virus neuraminidase inhibitors, target viral proteins. In recent years a novel approach has been developed: targeting cellular proteins to inhibit viral replication. As viruses are obligate intracellular parasites, they rely on cellular proteins for almost every step of their replication cycle. Such cellular proteins that are required by the virus represent alternative drug targets and it has been demonstrated that inhibition of such required factors can lead to inhibition of virus replication^5–7^. This new approach has several advantages, e.g. the number of drug targets is much higher than the limited number of viral proteins. Moreover, resistance to such inhibitors is less likely to occur as the inhibitor binds to a cellular rather than a viral protein^8^. Potential toxicities due to inhibition of cellular functions represent the main disadvantage of this strategy. In the case of IAV that causes an acute infection, a promising drug target would need to be required for efficient virus replication, but should be dispensable for the host for a few days to allow clearance of the virus from the respiratory epithelium.

A prerequisite for the development of such novel antivirals is detailed knowledge about the cellular proteins required by the virus. Genome-wide RNAi screens have greatly contributed to reveal such required cellular factors for IAV^5, 9–14^. A recent meta-analysis of data from different RNAi screens further helped to identify potential drug targets. Uniform bioinformatic analysis was applied to the different screening results and helped to uncover overlap between the screens and thereby reveal genes with evidence for a role as required host factor across multiple RNAi screens^15^. Among those genes were several members of the polo-like kinase (PLK) family, which are serine/threonine kinases highly conserved in eukaryotes and well known for their role in the regulation of cell cycle and cell division^16^. Of note, PLK1 has recently been identified as proviral host factor and potential drug target for hepatitis B virus^17^. Furthermore, PLK1 has been shown to down-regulate parainfluenza virus 5 gene expression by phosphorylating the viral P protein^18^. PLK1 also regulates hepatitis C virus (HCV) replication through hyperphosphorylation of NS5A^19^ and overexpression of FLAG-tagged PLK1 protein reduced the percentage of chikungunya virus infected cells in a reporter virus assay^20^. However, to date no proviral role for IAV has been reported for any of the polo-like kinases.

Here, we assessed their role as potential drug targets and found that knockdown of PLK1, PLK3, and PLK4, as well as inhibition with PLK inhibitors, reduced growth of IAV significantly. We also show that the PLKs are required during early stages of the viral replication cycle. Using a human lung explant system we demonstrate that inhibition of PLKs strongly limits virus replication in the absence of detectable toxicity. We therefore conclude that the PLKs represent promising new drug targets for the next generation of anti-influenza drugs.

## Results

**Figure 1:**
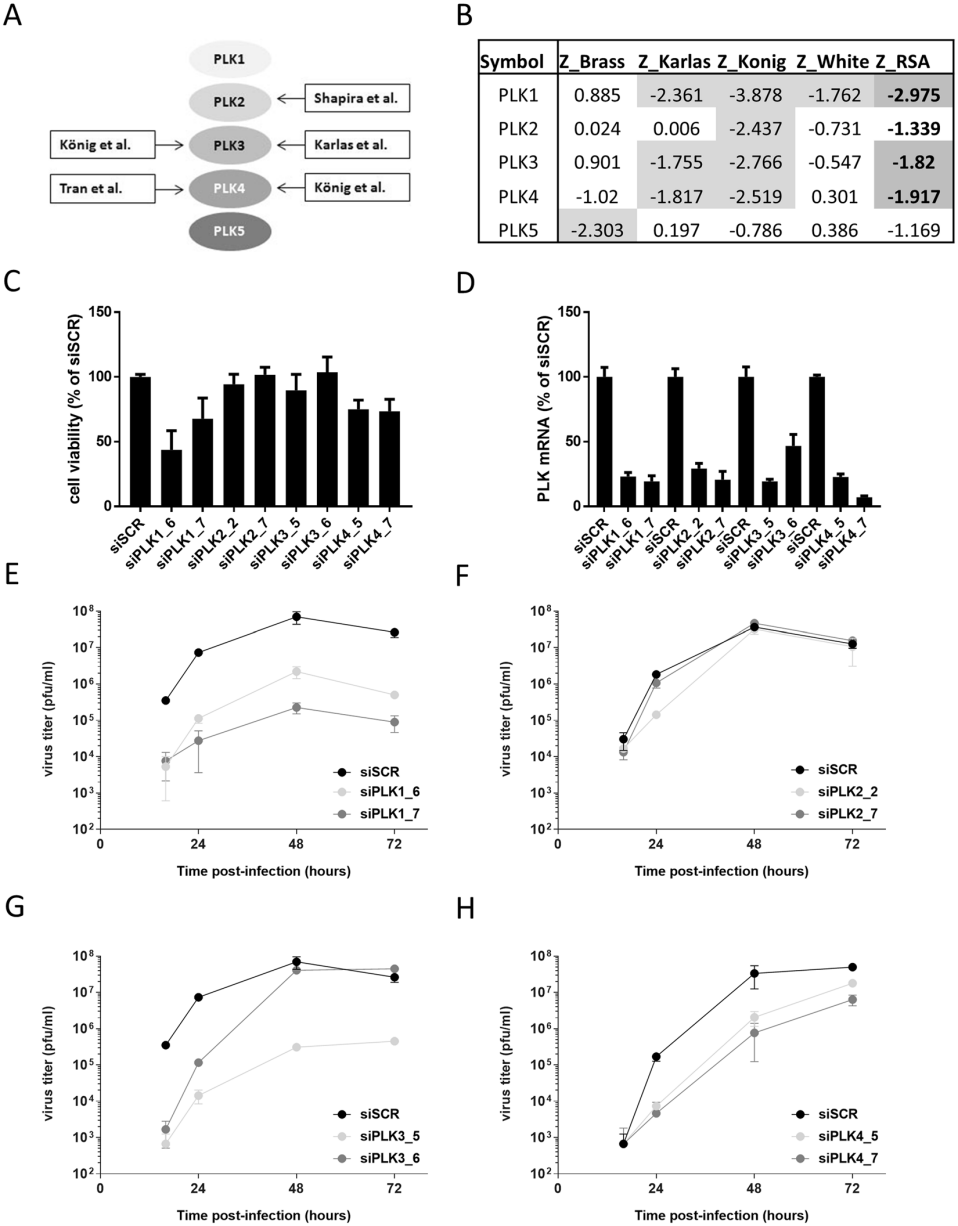
Knockdown of PLKs reduces viral growth. (**A**) Identification of PLKs as required host factors for IAV in different RNAi screens. Schematic drawing of the identification of different members of the PLK family as required host factors in RNAi screens. (**B**) Scores of the PLK family members in the meta-analysis of RNAi screens. (**C**) Cell viability. A549 cells were transfected with siRNAs targeting the indicated PLK genes or a control siRNA (siSCR). At 48 h post-transfection, cell viability was determined using the CellTiterGlo kit (Promega). Shown are averages from at least three independent experiments, each performed in triplicates, with standard deviation. (**D**) Knockdown efficiency. A549 cells were transfected with the indicated siRNAs and at 48 h post transfection cells were lysed, RNA extracted and expression levels of the indicated PLK genes were measured by RT-qPCR. (**E–H**) Virus titration. A549 cells were transfected with siRNAs targeting the indicated PLK genes or a control siRNA (siSCR). 48 h post-transfection, cells were infected with A/WSN/33 at MOI 0.01. At 16, 24, 48 and 72 h post-infection, tissue culture supernatants were harvested and titers determined by plaque assay. One representative experiment performed in triplicate is shown for each PLK family member; error bars indicate standard deviation. Please note that the siSCR samples in E and G are the same as these two experiments were performed in parallel.

Multiple genome-wide RNAi screens for host factors of influenza viruses have been performed, but the number of hits identified in two or more screens is limited^4, 8–13^. We noticed that members of the polo-like kinases (PLK) were among these common hits and therefore could represent potential drug targets for the inhibition of IAV. Analysis of the published hit lists of the different screens revealed that PLK2 was identified by Shapira *et al*.^11^, PLK3 scored as hit in the screens by König *et al*. and Karlas *et al*.^5, 9^ and PLK4 was listed as host factor of IAV by König *et al*. and Tran *et al*.^5, 14^ (Fig. 1A). Next, we investigated the performance of the different PLK family members in the meta-analysis of the RNAi screens. This meta-analysis introduced a uniform scoring system that enables easier comparison between screening results^15^. In addition to the scores in the different screens, an average score from all screens was provided for each gene (Z_RSA score). Generally, genes with Z_RSA scores of <−1.5 are considered hits that are required for efficient IAV replication. We found that PLK1, PLK3 and PLK4 could be classified as hits according to the meta-analysis (Fig. 1B). We therefore selected these genes for further analysis. We also included PLK2 as it had been identified as required host factor by Shapira *et al*.

In a first set of experiments we assessed the impact of knockdown of expression of the different PLKs on cell viability in the absence of virus infection (Fig. 1C). A549 cells were transfected with siRNAs targeting the different PLKs, or a scrambled control siRNA and cell viability was measured at 48 h post transfection. Depletion of PLK2 and PLK3 lead to very little impact on cell viability, however upon knockdown of PLK1 and, to a lesser extent PLK4, a reduction in viability was detected (Fig. 1C). We then confirmed that transfection of the siRNAs lead to reduced mRNA levels of the respective PLKs (Fig. 1D). In order to assess the impact of PLK levels on IAV infection, 48 h post siRNA transfection cells were infected with IAV strain A/WSN/33 at a low multiplicity of infection (MOI). Supernatants were harvested at different time points post infection and virus titers were determined by plaque assay. For PLK1 we observed a 10–100-fold inhibition of virus replication with both independent siRNAs at all time points examined (Fig. 1E). No consistent inhibition of viral replication was detected upon knockdown of PLK2 (Fig. 1F) but decreased PLK3 or PLK4 expression also lead to reduced viral titers (Fig. 1G,H). Overall, these results confirm the data from the RNAi screens, and establish PLK1, PLK3 and PLK4 as host factors required for efficient IAV replication. However, for PLK1 and PLK4, we cannot exclude that the inhibition of IAV replication is at least partially due to reduced cell viability.

**Figure 2:**
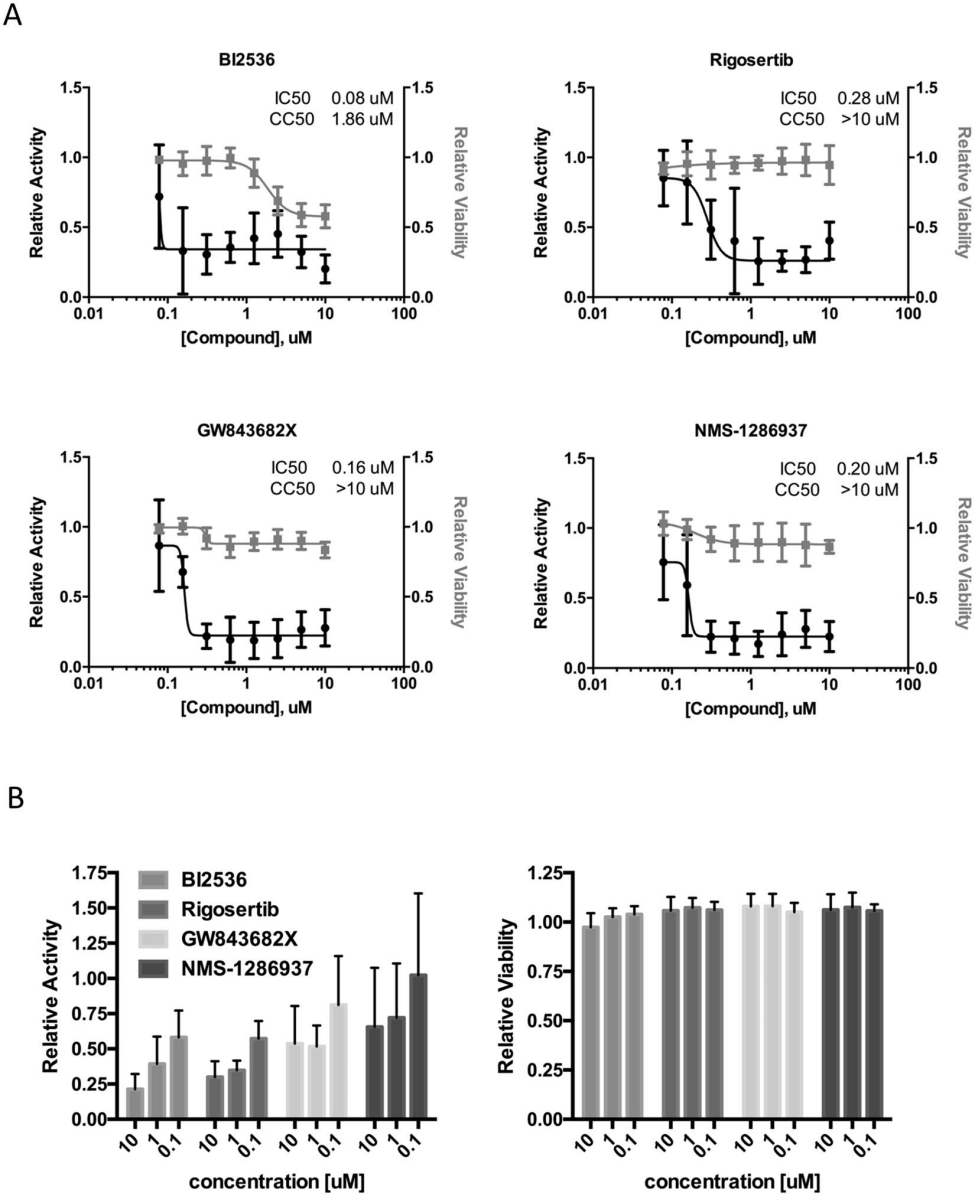
PLK inhibitors efficiently block IAV replication at non-toxic concentrations. The PLK inhibitors BI2536, Rigosertib, GW843682X and NMS-1286937 were used at different concentrations to investigate their effect on replication of recombinant influenza A WSN-Renilla luciferase virus (WSN-Ren). (**A**) PLK inhibitors block IAV in MDCK cells. MDCK-HA cells were pretreated with PLK inhibitors at the indicated concentrations for 2 h and subsequently infected with WSN-Ren at MOI 0.1 or mock treated for 30 hours in presence of inhibitors. Viral replication was assessed by luciferase activity relative to DMSO as control. Cell viability was determined using quantitation of ATP by luminescence relative to DMSO as control. Data were combined from three individual experiments with at least two replicates each. Shown are means with standard deviation. (**B**) PLK inhibitors block IAV in A549 cells. A549 cells were pretreated with PLK inhibitors at the indicated concentrations for 2 h, infected with WSN-Ren at MOI 3 or mock treated for 6 hours in the presence of inhibitors. Viral replication was assessed by measuring luciferase activity relative to DMSO as control. Data were combined from four experiments with two or more replicates each; shown are means with standard deviation.

Next, we tested four different inhibitors of PLKs (BI2536, Rigosertib, GW843682X and NMS-1286937) for their impact on IAV replication. All four inhibitors have been developed to primarily inhibit PLK1 but also display some activity against the other members of the family, especially PLK2 (BI2536, Rigosertib) and PLK3 (BI2536, GW843682X)^21–24^. For testing the inhibitors, a reporter virus was used in which the coding region of hemagglutinin (HA) was replaced by Renilla luciferase and the packaging sequences for the HA segment were added on either side (WSN-Ren). This reporter virus will produce mRNA encoding for Renilla luciferase instead of HA from the modified HA segment, and therefore has to be grown on HA-expressing cells^5^. MDCK-HA cells were pretreated with inhibitor for 2 h, and infected with the reporter virus at MOI 0.1 in the presence of the inhibitors for 30 h before luciferase was measured. In parallel, cells were treated with the inhibitor but kept uninfected and assayed for cell viability. We observed a dose-dependent inhibition of reporter activity for all four inhibitors tested with IC50s in the range of 80–280 nM (Fig. 2A). Importantly, for each compound, strong inhibition was observed at drug concentrations that displayed no or negligible effects on cell viability. This resulted in a selective index (SI) of 23.3 for BI2536, an SI of >35.7 for Rigosertib, an SI of >62.5 for GW843682X and an SI of >50 for NMS-1286937.

We also tested the inhibitors on human A549 cells using the WSN-Ren reporter virus. As these cells do not express HA, only single cycle infections can be observed. We pretreated cells with different concentrations of the inhibitors, infected them at MOI 3 and measured luciferase 6 h post infection. For BI2536 and Rigosertib we again detected a strong dose-dependent reduction of the luciferase signal (Fig. 2B left panel). For GW843682X and NMS-1286937 we also measured reduced luciferase values, but the effect was less pronounced compared to the other two inhibitors. Importantly, none of the inhibitors displayed toxic effects at the concentrations tested (Fig. 2B right panel). Overall, the inhibitor data further support the role of the PLKs as required host factors for IAV and establish them as potential drug targets.

**Figure 3:**
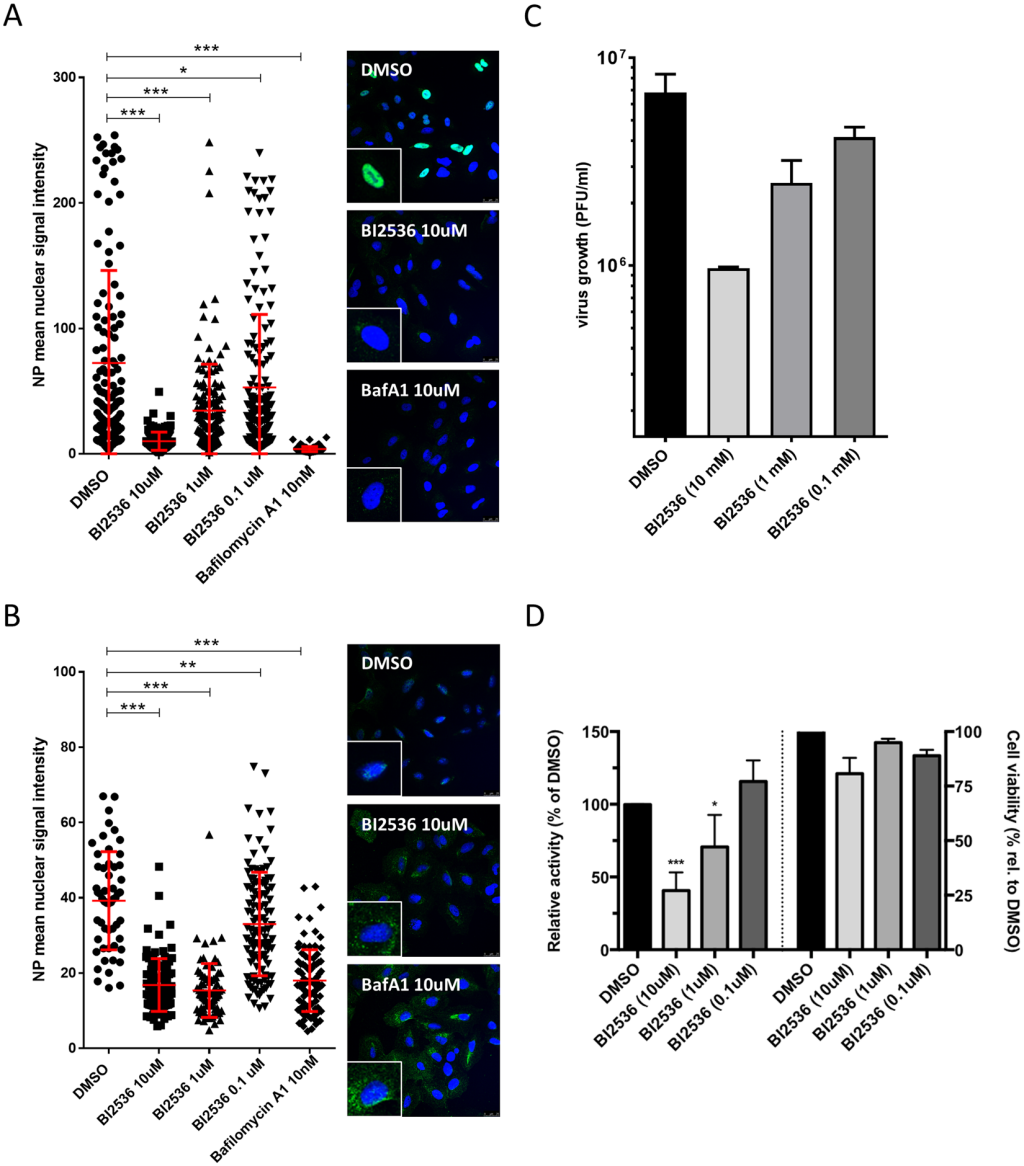
PLKs are required early in the infectious cycle. (**A**) Inhibition of PLKs leads to decreased nuclear NP signal at 3 h post infection. A549 cells were treated with indicated amounts of BI2536 or DMSO for 2 h. Cells were infected with WSN at MOI 10 for 1 h on ice with inhibitor/DMSO present in the tissue culture medium. After infection, cells were washed and then incubated with inhibitor-containing medium for 3 h at 37 °C. Then, cells were fixed and stained for NP (green) and DNA (blue). Images were acquired on a Leica SP5 confocal microscope and NP mean nuclear signal intensity was quantified using ImageJ software. Shown is one representative experiment out of three independent experiments; error bars indicate standard deviation. Statistical significance of differences between DMSO and the inhibitors was determined using the Mann Whitney test (*P < 0.05, ***P < 0.001). (**B**) Inhibition of PLKs leads to decreased nuclear accumulation of incoming vRNPs. Same experimental set-up as in (**A**) but CHX (100 mg/ml) was present in the tissue culture medium. Statistical significance of differences between DMSO and the inhibitors was determined using the Mann Whitney test (**P ≤ 0.005, ***P < 0.001). (**C**) Inhibitor treatment early in infection reduces IAV replication. A549 cells were pre-treated with inhibitor-containing medium at the indicated concentrations for 1 h at 37 °C. Then, cells were infected with A/WSN/33 at MOI 1, diluted in inhibitor-containing medium for 1 h at 37 °C. After infection, cells were washed and treated with inhibitor-containing medium for additional 2 h at 37 °C. Then, cells were kept in inhibitor-free medium for 14 h. At 17 h pi, tissue culture supernatants were harvested and viral growth was determined by plaque assay. Shown are averages of three independent experiments, each performed in duplicates, with standard deviation. (**D**) Inhibition of PLKs impacts IAV polymerase activity. HEK293T cells were used for IAV RNP reconstitution. Six hours after transfection, medium was changed and DMSO or the indicated concentrations of BI2536 were added. After 18 hours cells were harvested and luciferase activities were assayed (left Y-axis). In parallel, untransfected cells were treated with DMSO or the indicated concentrations of BI2536 and after 18 hours cell viability was tested (right Y-axis). Three independent experiments with three replicates each were performed and the mean with standard error of the mean is shown. Statistical significance of differences between DMSO and the different BI2536 concentrations was determined using the Student’s *t* test (*P < 0.05, ***P < 0.001).

In a next series of experiments, we aimed to determine the step of the viral replication cycle that is dependent on PLKs using the inhibitor BI2536, a peptide with a high specificity for PLK1 that targets the ATP-binding pocket of the PLK kinase domain^25^. This molecule was selected because it displayed the lowest IC50 in MDCK cells, and gave the strongest reduction in A549 cells in the reporter virus system. The single cycle experiments in A549 cells already suggested that early steps, such as virus entry, viral transcription and replication or translation of viral proteins are dependent on PLK rather than later stages, like assembly or budding. In order to confirm the impact on early stages, we pretreated A549 cells with the inhibitor for 2 h, infected in the presence of the inhibitor for 1 h and then let the infection proceed for 3 h in the presence of the inhibitor. Cells were fixed, stained for nuclei and viral NP and analyzed by confocal microscopy. In control cells treated with the inhibitor vehicle only, we observed strong green staining in the nuclei of the infected cells indicating viral replication. This nuclear NP signal was absent when a positive control inhibitor, Bafilomycin A1^26^, was used (Fig. 3A). In BI2536-treated samples we detected a statistically significant dose-dependent inhibition of the nuclear NP signal, confirming the impact of PLK inhibition on early stages of infection. When we repeated the experiment in the presence of cycloheximide, a strong inhibitor of protein synthesis, we also observed a statistically significant inhibition of nuclear NP accumulation (Fig. 3B). As cycloheximide blocks the production of new NP, only incoming RNPs are detected in this assay and the overall signals are weaker than in the absence of cycloheximide as indicated by the lower fluorescence intensity^7, 27^. Therefore, we can conclude that inhibition of PLKs impacts entry of IAV resulting in reduced amounts of imported vRNPs within the nucleus. To further verify this finding, we performed an IAV infection using strain A/WSN/33 and added the PLK inhibitor only early during infection. We pretreated A549 cells with BI2536 for 1 h, infected in the presence of the inhibitor for 3 h, before changing to regular infection medium. At 17 h post infection, supernatants were harvested and virus titers were determined by plaque assay. We observed a dose-dependent inhibition of virus growth despite the inhibitor being only present in the first 3 h of infection (Fig. 3C). Consistent with previous results, these data suggest that the inhibitor acts on virus entry and/or viral transcription and production of viral proteins.

In addition, we tested the effect of PLK inhibition in an IAV polymerase assay. Towards this aim, expression plasmids for the different components of the IAV polymerase and viral NP were co-transfected with a reporter construct that allows for the measurement of IAV polymerase activity. At 6 h post transfection, different concentrations of BI2536 were added and 18 h later reporter activity was measured. In parallel, cell viability was determined. Polymerase activity was reduced by approximately 60% at the 10 uM concentration and about 30% for the 1 uM concentration but no inhibition could be seen for the lowest inhibitor concentration (Fig. 3D left panel). Only small effects on cell viability were measured (Fig. 3D right panel) indicating specific inhibition of IAV polymerase activity by BI2536. In sum, these results show that PLKs are required early in IAV infection, in particular during the entry and RNA replication stages.

In order to evaluate whether the members of the PLK family could represent potential drug targets for IAV in a relevant primary culture system, we employed an *ex vivo* human lung tissue model. This allowed us to measure the impact of the PLK inhibitor on IAV replication in authentic and physiologically relevant human lung tissue cultures.

**Figure 4:**
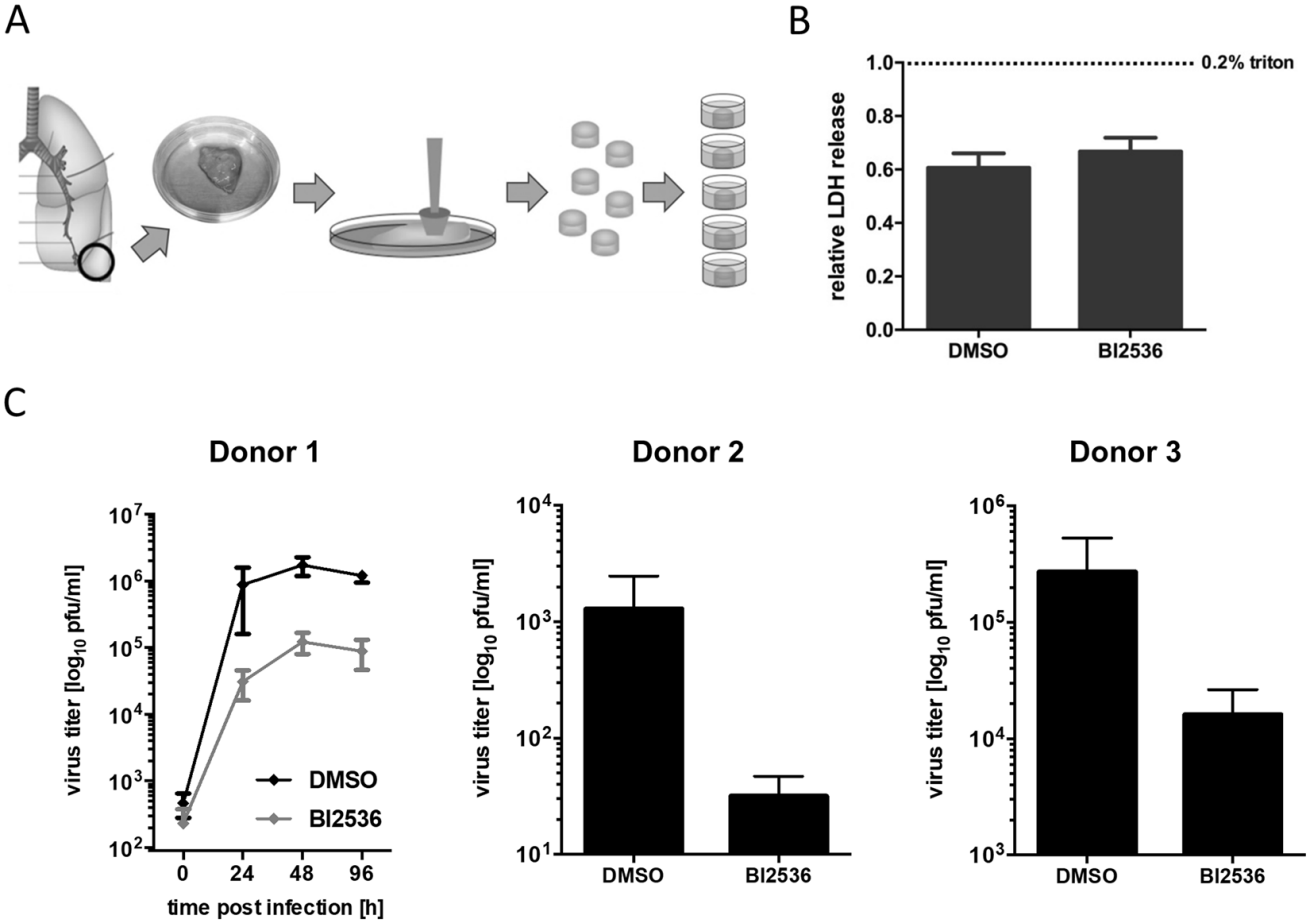
BI2536 inhibits IAV replication in *ex vivo* human lung tissue. (**A**) Schematic overview of the *ex vivo* human lung tissue model. Tissue samples were provided by the Cooperative Human Tissue Network which is funded by the National Cancer Institute. Tissue was stamped into small cylinders (diameter of 4 mm), which were incubated individually in primary cell media in 96-well plates. (**B**) PLK inhibition displays no toxicity in the *ex vivo* human lung tissue model. Tissue samples were treated with 10 uM BI2536, DMSO or 0.2% triton for 48 h. Lactate dehydrogenase (LDH) activity in supernatants was measured and LDH activity at 0.2% triton was set as 1. LDH activity after DMSO or compound treatment was determined relative to the positive control. Four independent experiments with three replicates each were performed and the mean with standard error of the mean is shown. (**C**) PLK inhibition blocks IAV replication in the *ex vivo* human lung tissue model. Tissue samples were infected with 1 × 10^5^ pfu A/Panama/2007/1999 and treated with 10 uM BI2536 or DMSO post infection. Supernatants were collected after 24, 48 and 96 h in case of donor 1 and after 48 h for donors 2 and 3. Viral titers were determined by plaque assay in MDCK cells. Three independent experiments with three or more replicates each were performed and the mean with standard error of the mean is shown.

Figure 4A displays a schematic drawing of the preparation of the lung organ cultures, which represent tumor-free tissue derived from the distal parts of human lungs. First, we treated the lung cultures with the PLK inhibitor BI2536 ﻿at 10 uM﻿ for 48 h and measured LDH release as a measure for toxicity^28–30^, including detergent treatment as positive control. Remarkably, no toxicity was observed upon inhibitor treatment for 48 h (Fig. 4B). Next, we infected the lung cultures with the seasonal H3N2 IAV strain A/Panama/2007/1999 for 1 h before changing to medium containing the inhibitor. Supernatants were collected from the lung cultures at different time points post infection and virus titers were determined by plaque assay. The H3N2 isolate was chosen for this analysis as previous studies had indicated that H3N2 strains grow to higher titers in the lung culture model than H1N1 viruses^31, 32^. We observed a strong reduction of viral replication, and this inhibition of IAV was consistent for lung samples derived from three different human donors (Fig. 4C). We conclude that treatment with PLK inhibitor BI2536 at this concentration does not induce toxicity but can potently inhibit IAV replication in human lung organ cultures.

## Discussion

Recent genome-wide RNAi screens have identified a plethora of cellular factors required for efficient IAV replication with the long-term goal of finding alternative drug targets for the development of the next generation of antivirals against influenza^33, 34^. However, thus far the potential of these data has not been fully exploited as only few follow-up studies have been performed to reveal the role of the identified factors in the IAV replication cycle on a molecular and cell biological level. Here, we studied the polo-like kinases that had emerged as promising candidates for follow-up studies from the RNAi screens. We found that several members of the PLK family were identified as hits in different screens, and this was confirmed by a recent meta-analysis of the RNAi screens highlighting the potential of the PLKs as possible drug targets. We could show that individual knockdown of PLK1, PLK3 and PLK4 indeed resulted in reduced IAV titers as predicted from the meta-analysis. Treatment of cells with four different PLK inhibitors also resulted in significant inhibition of virus replication. For the most potent inhibitor, BI2536, we could further demonstrate that inhibition occurs at early stages of the replication cycle, with the strongest impact on entry and an additional effect on polymerase activity. Importantly, when testing the PLK inhibitor in a human lung tissue model, we also observed potent inhibition of IAV replication at inhibitor concentrations that displayed no toxicity in this state-of-the-art 3D system. With these data, we not only establish the PLKs as potential drug targets for the development of antivirals for influenza, but also suggest to consider the PLKs as potential pan-viral drug target. In particular PLK1 has also been studied in the context other viruses and a proviral role of PLK1 could be demonstrated for hepatitis C and hepatitis B virus infections^17, 19^. Thus, PLK inhibitors could have broad antiviral activity.

PLKs execute a variety of functions within the cell. Besides their multiple well-studied roles in cell cycle regulation, PLKs are involved in other crucial cellular functions such as the regulation of various survival and stress responses. IAV likely depends on PLK-mediated regulation of cellular growth and stress processes and may benefit from both, growth stimulatory and –inhibitory effects mediated through PLK activity. We hypothesize that a link between IAV and PLKs might be p53 which fulfills a central role in apoptotic signaling, and is well-known for its role as tumor suppressor gene^35^. PLKs have been shown to regulate p53 expression and activity and in turn appear to be regulated by p53^25, 36, 37^. Thereby, PLKs play a critical role in the determination of cell fate in response to stress, which is likely to have a large impact on viral replication. Furthermore, IAV is known to directly modulate pro- and anti-apoptotic signaling and IAV has been shown to activate p53 in a bi-phasic manner: while virus replication was shown to be required for p53 activation during later stages of infection, early events during virus entry such as adsorption were shown to be sufficient to activate the tumor suppressor shortly after infection^38^. There is currently no consensus on whether activation of stress signaling cascades and apoptosis is beneficial or detrimental for IAV growth, and it is likely that the timing and extent of activation of such pathways is critical for efficient virus replication. One could speculate that IAV depends on PLK activity to counteract premature activation of stress, antiviral, and apoptotic signaling pathways, and to push through cell cycle checkpoints in order to establish productive infections. This is consistent with earlier studies suggesting that the extent of apoptosis in a cell line inversely correlates with the permissiveness to IAV infection^39^. PLKs might be required to stimulate cell survival and thereby reduce early immune recognition through premature activation of antiviral and proapoptotic pathways, for example through the IAV-induced inhibition of authophagy: IAV has been shown to directly inhibit autophagy which in turn increases apoptosis and viral antigen release^40^. Interestingly, treatment with BI2536 has been demonstrated to block autophagy and to induce accumulation of LC3-positive punctae, which increases cell death similarly to IAV infection^41^. This might indicate that at early stages of infection, inhibition or knockdown of PLK activity might accelerate several proapoptotic/antiviral processes leaving too little time to complete virus replication. The precise roles of PLKs during the viral life cycle remain to be identified, but it is tempting to speculate that PLK function is required by IAV to finetune apoptotic and antiapoptotic signaling cascades, thereby creating an optimal environment for viral replication in a time-dependent manner.

When considering a cellular protein as drug target it is crucial to study possible effects on cell viability in addition to the antiviral potential. In contrast to antivirals for chronic virus infections, such as HIV, only short-term effects have to be considered in the case of influenza, as it is an acute infection that lasts only for a few days. Furthermore, as the infection is limited to the respiratory tract, systemic delivery may not be required, thus limiting potential toxicities. Nevertheless, one would assume that inhibitors against cellular factors display higher toxicity than antivirals designed against viral components, and therefore high priority has to be given to toxicity testing if developing inhibitors against cellular factors, such as the PLKs. In line with previous reports we observed a significant reduction of cell viability upon knockdown of PLK1 and PLK4^42, 43^, but not for PLK2 and PLK3. For all four PLK inhibitors tested, a broad range of concentrations could be identified that displayed potent virus inhibition but no impact on cell viability. In the human lung tissue culture model we evaluated the highest non-toxic concentration used in the standard cell lines and saw no effect on viability. These data suggest that there may be a therapeutic window for the use of PLK inhibitors as antivirals.

Importantly, the PLKs have emerged as promising drug target in cancer research as PLK levels are increased in a number of tumors and high expression has been linked to reduced survival rates^21, 44, 45^. PLK inhibitors, including BI2536, have already been tested in clinical trials and were found to be safe in the tested patient groups^46–52^. Such data on safety and bioavailability will be helpful to further evaluate the potential of PLK inhibitors for treatment against influenza.

Of note, most of the PLK inhibitors currently in clinical trials have been developed as inhibitors of PLK1^45, 49^, as it is the best studied member of the family. As we not only observed inhibition of viral replication for PLK1 knockdown but also for PLK3 and PLK4 downregulation by siRNAs it would be desirable to develop inhibitors specific for the other PLK members. In future studies, such inhibitors could be tested for their impact on virus replication, in particular in the human lung tissue culture model that is not amenable to siRNA transfections.

In summary, we have demonstrated that different members of the PLK family are required host cell factors for efficient IAV replication, and represent promising drug targets for the development of the next generation of antivirals.

## Methods

### Cells, viruses, and compounds

A549, HEK293T and MDCK cell lines were maintained in DMEM containing penicillin/streptomycin (Life Technologies) supplemented with 10% FCS. Influenza virus strain A/WSN/33 was grown in A549 cells; strain A/Panama/2007/1999 in MDCK cells and the reporter virus WSN-Renilla was propagated on MDCK-HA cells as described previously^5^. All influenza virus stocks were titered by plaque assay using MDCK or MDCK-HA cells. The following compounds were used: Bafilomycin A1 (Sigma-Aldrich), cycloheximide (Sigma-Aldrich), BI2536 (Selleck Chemicals), Rigosertib, GW843682X and NMS-1286937 (MedChemExpress). According to the manufacturers, the PLK inhibitors demonstrate the following IC50 values: BI2536: PLK1 0.83 nM, PLK2 3.5 nM, PLK3 9.0 nM; Rigosertib: PLK1 9.0 nM, PLK2 270 nM; GW843682X: PLK1 2.2 nM, PLK3 9.1 nM; NMS-1286937: PLK1 2.0 nM.

### siRNA transfection and cell viability assay

siRNA transfections were performed as described previously^7^. In brief, A549 cells were transfected in suspension with 30 nM siRNA (Qiagen) diluted in Opti-MEM (Life Technologies) using the RNAiMAX reagent according to the manufacturer’s protocol (Invitrogen). At 48 h post-transfection, cells were either infected or cell viability was determined using the CellTiter-Glo assay (Promega). In addition, the CellTiter-Glo kit was used to assess the effect of inhibitor treatment on A549 and HEK293T cells.

### RT-qPCR

At 48 h post-siRNA transfection, total RNA was extracted from A549 cells (2 × 10^5^ cells/24-well) using RNeasy Mini Kit (Qiagen). The complementary DNA (cDNA) synthesis was performed with 1 µg of RNA per sample, using the Super Script III reverse transcriptase (Invitrogen) according to manufacturer’s protocol. RT-qPCRs were performed in 20 µl using Fast Plus EvaGreen qPCR Master Mix (Biotium) in an Applied Biosystems 73000 RT-PCR System thermocycler. GAPDH mRNA was used as control for normalization of the amount of RNA. The following primers were used: PLK1-FW: CACCAGCACGTCGTAGGATTC; PLK1-RV: CCGTAGGTAGTATCGGGCCTC; PLK2-FW: GGGACTCTTGGCAGCTGTAG; PLK2-RV: TTGGTGACCCACTGAAATGA; PLK3-FW: TTTTCGCACCACTTTGAGGAC; PLK3-RV: GAGGCCAGAAAGGATCTGCC; PLK4-FW: CCTTATCACCTCCTCCTTC; PLK4-RV: CCAAGTCCTTCATTTGTAACC; GAPDH-FW: CATCACGCCACAGTTTCCCGG GAPDH-RV: CTGGCGTCTTCACCACCATGG.

### Virus infection

Cells were washed once with phosphate buffered saline (PBS) and then infected with the respective amount of virus diluted in PBS supplemented with 0.02 mM Mg^2+^, 0.01 mM Ca^2+^, 0.3% bovine serum albumin (BSA), and 1% penicillin-streptomycin (infection PBS) for the siRNA experiments and in DMEM containing 0.3% BSA, 20 mM HEPES, and 1% penicillin-streptomycin for the inhibitor experiments. Infection was performed at 37 °C for 1 h. Cells were washed with PBS and then maintained in DMEM containing 0.3% BSA, 20 mM HEPES, and 1% penicillin-streptomycin (post infection DMEM). Following harvest of tissue culture supernatants of infected cells, virus titers in the supernatants were determined by standard plaque assay on MDCK cells. For the immunofluorescence experiments, A549 cells were infected with A/WSN/33 on ice for 1 h to synchronize infection. Then, cells were shifted to 37 °C for 3 h to allow infection to proceed.

### PLK inhibitor assay

MDCK cells stably expressing hemagglutinin (MDCK-HA) or A549 cells were cultured to 80–90% confluency, washed with phosphate-buffered saline (PBS, Life Technologies), trypsinized with 0.05% Trypsin-EDTA (Life Technologies), and resuspended in 1X Dulbecco’s Modified Eagle Medium (DMEM, Life Technologies) supplemented with 10% fetal bovine serum (FBS, Life Technologies), 1% penicillin/ streptomycin (P/S), and 0.15% sodium bicarbonate (Life Technologies). The cells were then pelleted and resuspended in DMEM containing 1% FBS, 0.3% bovine albumin (Sigma), 20 mM HEPES, and 1% P/S. Per well 90 μl of the diluted cells were dispensed into 96-well plates (20,000 MDCK-HA or 30,000 A549 cells per well) and incubated at 37 °C, 5% CO_2_ overnight. Compounds were diluted in the same media as cells and DMSO was used as control. Compound dilutions were added to each well (10 μl), and plates were incubated at 37°C, 5% CO_2_ for 2 hours. Next, the cells were infected with 25 μL of recombinant influenza A WSN-Renilla luciferase virus (WSN-Ren)^5^, at MOI 0.1 in case of MDCK-HA or MOI 3 for A549 cells and incubated at 37 °C, 5% CO_2_. Thirty hours (MDCK-HA) or six hours (A549) post-infection, 60 μL of Renilla-Glo (Promega) were added to each well and Renilla luciferase activity was measured with an EnSpire Alpha Plate Reader (Perkin Elmer). In order to test cell viability, plates were treated as described above but instead of infecting with WSN-Ren, medium without virus was added to the cells and cell viability was tested using the CellTiter-Glo Luminescent Cell Viability Assay kit (PROMEGA) according to manufacturer’s recommendations.

### Immunofluorescence microscopy

In order to detect early viral replication we used the following assay which measures nuclear NP signals at 3 h post infection in the presence or absence of cycloheximide, a protein synthesis inhibitor. In this system, diffuse NP signals from incoming virus are detected in the cytoplasm and the nucleus in the presence of cycloheximide. In the absence of cycloheximide much stronger NP signals derived from newly produced NP are detected mostly in the nucleus. For NP staining, cells were fixed with 3.7% paraformaldehyde (PFA), permeabilized with 0.5% Triton-X-100 and blocked with 2% bovine albumin all diluted in PBS. Cells were incubated with mouse monoclonal anti-NP (HB65 hybridoma supernatant). Alexa488-labelled donkey anti-mouse IgG-specific secondary antibody was used (Life Technologies). Nuclei were visualized using DAPI (Life Technologies). For mounting, Slowfade Gold antifade mounting medium with DAPI (Life Technologies) was used. Images were acquired with a Leica SP5 confocal laser-scanning microscope. Image processing was performed using LAS AF lite (Leica) and ImageJ software.

### Minireplicon assay

The recombinant minireplicon assay was performed essentially as described previously^53^. In brief, cultures of HEK293T cells (2 × 10^5^ cells/well in 6-well culture plates) were transfected with a mixture of plasmids expressing the RNP components (pCMVPA, 25 ng; pCMVPB1, 125 ng; pCMVPB2, 125 ng; and pCMVNP, 500 ng) and a genomic plasmid expressing a viral RNA (vRNA)-like Firefly luciferase reporter gene (pPolNS-FF-Luc, 500 ng). A plasmid expressing Renilla luciferase (pCMV-Renilla-Luc, 100 ng) was cotransfected as an internal transfection control. Six hours after transfection, media was changed for DMEM 10% FBS plus DMSO or different concentrations of the BI2536 compound (0.1, 1 and 10 uM). At 24 hours post-transfection, cells were harvested and Firefly and Renilla luciferases activities were assayed with a Dual-Glo Luciferase Assay System kit (PROMEGA) according to manufacturer’s recommendation. Firefly luciferase measures were normalized using Renilla luciferase measures. DMSO average was taken as 100% and all samples were referred to it.

### IAV infection of *ex vivo* human lung tissue

Tissue samples were provided by the Cooperative Human Tissue Network which is funded by the National Cancer Institute. Tumor-free human lung tissue from the distal parts of the lung was stamped into small cylinders (diameter of 4 mm) and incubated in 96-well microtiter plates in 250 μl primary cell medium (Airway Epithelial Cell Basal Medium with Bronchial Epithelial Cell Growth Kit, both ATCC) at 37°C with 5% CO_2_. After overnight incubation, lung organ cultures were inoculated with 1 × 10^5^ pfu A/Panama/2007/1999 in 100 μl media for 1 h. Next, each lung tissue sample was moved to 250 μl fresh medium containing either the BI2536 compound at a final concentration of 10 uM or DMSO as a control. Viral titers in supernatants collected at different time points post infection were determined by plaque assay on MDCK cells.

### Toxicity assay in *ex vivo* human lung tissue

Tissue samples were treated with 10 uM BI2536 compound, DMSO or 0.2% triton for 48 h. Standard lactate dehydrogenase (LDH) cytotoxicity detection assay (Roche) was performed with supernatants (100 μl) according to manufacturer’s recommendations. For data analysis, LDH activity at 0.2% triton was used as positive control and set as 1. Toxicity was measured as LDH activity relative to positive control.

All experiments using human lung tissue samples were carried out in accordance with relevant guidelines and regulations according to the Cooperative Human Tissue Network (www.chtn.org). The experimental protocols were approved by the Sanford Burnham Prebys Medical Discovery Institute Institutional Review Board (IRB). Informed consent was obtained from all subjects.

### Data availability

The datasets generated during and/or analysed during the current study are available from the corresponding author on reasonable request.
